# Older Adult Volunteers’ Experiences Delivering a Lay-Led Behavioral Activation Program for Depression Among Community-Dwelling Older Adults: A Mixed Methods Study

**DOI:** 10.1016/j.osep.2025.05.001

**Published:** 2025-06-09

**Authors:** Nicole O. Crawford, Lesley Steinman, Isabel Rollandi, Brenden Li, Enid Rubio, Brittany Mosser, Julien Rouvere, Brittany E. Blanchard, Amber Gum, Jo Anne Sirey, Patrick J. Raue

**Affiliations:** Department of Mental Health Law and Policy (NOC, AG), University of South Florida, Tampa, FL; Department of Health Systems and Population Health (LS), Health Promotion Research Center, University of Washington School of Public Health, Seattle, WA; Weill Cornell Institute of Geriatric Psychiatry (IR, JAS), Weill Cornell Medicine, New York, NY; Morsani College of Medicine (BL), University of South Florida, Tampa, FL; Brandon Senior Center (ER), Tampa, FL; and the Department of Psychiatry and Behavioral Sciences (BM, JR, BEB, PJR), University of Washington School of Medicine, Seattle, WA.

**Keywords:** Community-based organizations, depression, lay delivery, volunteers, mixed methods, implementation outcomes

## Abstract

**Objectives:**

To evaluate coaches’ experiences delivering a lay-led depression intervention (Do More, Feel Better; DMFB) during a Type I hybrid effectiveness-implementation RCT.

**Methods:**

We conducted a process evaluation to assess DMFB feasibility, appropriateness, fidelity, and acceptability in 12 senior centers in Florida, New York, and Washington (USA). DMFB is streamlined Behavioral Activation delivered by older adult volunteers from senior center communities. Data sources included eligibility and exit interviews with surveys and external fidelity ratings of session recordings.

**Results:**

The study sample included 45 older adults (M_age_ = 69.8, SD_age_ = 6.3), 85.3% women, 45.4% people of color, 24.1% low-income, 53.1% live alone. Feasibility: Of 66 individuals interested in serving as DMFB lay coaches, 45 (68.2%) were eligible, 34 (51.2%) were trained, 27 (40.1%) were certified, and 24 (36.4%) engaged 1 or more clients. Appropriateness: Eligible older adult volunteers had high capacity to serve as coaches (M = 5.3, SD = 0.8, 6-pt scale), bringing lived experience helping others, managing their mental health, good communication and organizational skills, and valuing volunteering. Fidelity: External global ratings were “very good” (M = 4.6, SD = 0.7, 5-pt scale); areas for improvement were managing time for PHQ-) administration and activity scheduling, and need for more person-centered communication. Acceptability: Coaches rated high confidence delivering DMFB (M = 4.7, SD = 0.6, 5-pt scale) and that they received adequate training (M = 4.5, SD = 0.6, 5-pt scale) and supervision (M = 4.6, SD = 0.6, 5-pt scale). Coaches found satisfaction helping others, connecting with clients, and applying DMFB to their mental health.

**Conclusions:**

This study adds to limited literature on implementation outcomes for lay-led community-based interventions for older adults. Preliminary findings document successful implementation.

## OBJECTIVES

One in six people will be over the age of 60 by 2030.^1^ To support healthy aging, alternative options for mental health care are needed. Prevalence estimates suggest over 31% of adults 60 and older experience some level of depression.^2^ Depression often goes undiagnosed and undertreated in older adults, due to lack of access to care, cost, stigma, ageism, racism, and other systemic inequities,^3^ as well as workforce shortages contributing to a lack of adequate clinical services. Over 150 million people live in mental health shortage areas, also called service deserts, where access to behavioral health providers including psychiatrists and counselors is limited.^4^ Social determinants of health, isolation, and preferences for unavailable psychosocial treatments can further compound the mental health burden faced by older adults living with depression.^5^

Community-based interventions offer one path for improving access to quality mental health care. Task shifting from clinicians to trained community providers (e.g., volunteers, community health workers) can address the widespread problem of mental health care workforce shortages.^6,7^ Senior centers represent promising service settings in which to offer acceptable care. Across the United States, over one million older adults are served by 11,000 senior centers.^8^ Senior centers are part of the national aging service networks that support older adults aging in place (independently). They provide important services that address social determinants of health like housing and social support^5^ for the many older adults living longer yet with limited incomes.^9^ Senior centers offer nutrition programs, health and wellness programming, case management, and recreational activities.

Senior centers provide older adults with diverse opportunities to volunteer within the centers and through other community connections. Approximately one in three older adults volunteer, with 22 million older adults volunteering 2.2 billion hours in 2018.^10^ Volunteering can increase individuals’ physical activity, self-esteem, and self-efficacy, and decrease isolation and depression.^11,12^ Older adult volunteers provide value to the communities they serve and, with appropriate training in evidence-based programs, may be one solution to address the mental health workforce shortage. Lay-delivered interventions may be equally or more acceptable to older adults and may show similar effectiveness in improving clinical outcomes.^13−15^ In addition, training older adults to deliver lay interventions that support mental wellness also offers potential for improving their own mental health.^16−18^

In response to large numbers of senior center clients who live with untreated depression and the dearth of geriatric mental health providers, our research team partnered with senior centers to streamline Behavioral Activation (BA) to match the skill set of older volunteers (“Do More, Feel Better”; DMFB). The lay-delivery model utilizes existing volunteer resources that can address the insufficient workforce and has potential for being an acceptable and sustainable delivery model.^13−15,19^ We are currently testing the effectiveness of the DMFB lay coach model in increasing activity level and reducing depressive symptoms in a multi-site, noninferiority randomized controlled trial (RCT).^6^

This RCT also offers an opportunity to evaluate DMFB delivery by lay coaches. Effective implementation is typically gauged by evaluating implementation outcomes—intermediate outcomes which can impact the success of delivering an intervention in a specific context.^20^ These include constructs like feasibility of implementation, appropriateness of program fit in different populations, providers, and settings, fidelity to delivering the program as intended, and acceptability of the program to providers and participants.^21^ A recent review^20^ of the Implementation Outcomes Framework (IOF) found that only 6.3% and 8.3% of implementation outcomes studies to date have been conducted in social service settings and with older adults, respectively. As such, implementation scientists may inadvertently perpetuate gaps in mental health care by not engaging with populations and in settings that are recommended to improve mental health equity for older adults.^3^ It is particularly important to engage interventionists’ voices in research as key ingredients for implementation success.^22^ As such, we conducted implementation research as part of our effectiveness RCT to understand the feasibility, appropriateness, and acceptability of DMFB from coaches’ perspectives, as well as their fidelity to the intervention based on external expert ratings.

## METHOD

### Design

We conducted an exploratory process evaluation during our ongoing multi-site RCT to evaluate the effectiveness of Do More, Feel Better (DMFB).^6^ We used a parallel mixed methods design to evaluate early-stage implementation outcomes, simultaneously collecting quantitative and qualitative data to evaluate coaches’ experiences delivering DMFB.^23,24^ This study was reviewed and approved by our UW Institutional Review Board (#11434).

### Intervention

DMFB is a streamlined manualized version of Behavioral Activation (BA), an established psychotherapy with strong evidence for treating depression among older adults from diverse backgrounds.^25^ BA counters the tendency for people with depression to withdraw, be inactive, and isolate, through planning and engaging in meaningful and rewarding activities. DMFB is delivered in nine weekly 45−60-minute phone, video-conferencing, or in-person sessions with trained lay coaches − older adult volunteers from senior centers and other community-based settings.^19^ DMFB clients meet weekly with their coach to choose and reflect on activities that might help to boost their mood. DMFB implementation strategies^26^ include capacity-building strategies to support older adults to deliver DMFB (training, technical assistance, and clinical supervision) and integration strategies to embed DMFB in social service settings.^27^

### Setting and Participants

The study was conducted in three U.S. locations: Hillsborough County, Florida; New York City, New York, and the Seattle metro area, Washington. In 2021−2023, we recruited twelve senior centers as study sites, who partnered with our research teams to engage older adult volunteers as DMFB coaches. Volunteers were recruited via various outreach strategies (e.g., word of mouth, presentations, newsletters) and screened for eligibility using a structured interview.

Volunteers were eligible for training to become coaches if they were age 60 or older; met criteria for good interpersonal and organizational skills (research staff rating of open-ended interview questions), emotional stability (Patient Health Questionnaire-9^28^ [PHQ-9] < 10), and cognitive functioning (6-Item Memory Cognition Screen^29^); and did not have experience as a clinical mental health professional. Older adult senior center clients were eligible if they had clinically significant depressive symptoms (PHQ-9≥10 and Hamilton Depression Scale^30^ [HAM-D]≥10) and were ineligible if they were experiencing active suicidal ideation, had a diagnosis of bipolar disorder, psychotic-spectrum disorder, dementia, or other significant cognitive impairment (Telephone Interview for Cognitive Status-Modified^31^ [TICS-M]<21), or were in weekly psychotherapy or on antidepressants unless taking stable doses for ≥12 weeks.^6^

### Data Collection

#### Data sources:

Data were collected from potential volunteer coaches via eligibility interviews during the preparation phase and exit interviews during the implementation phase after coaches delivered DMFB with up to five clients. These 30−45 minute interviews included both open-ended qualitative questions and close-ended quantitative questions (Supplementary 1). Trained research staff conducted interviews by telephone or video-conferencing, and all coaches were invited to participate. We used REDCap^32^ for data collection and management.

#### Implementation outcomes:

**FIGURE 1.**
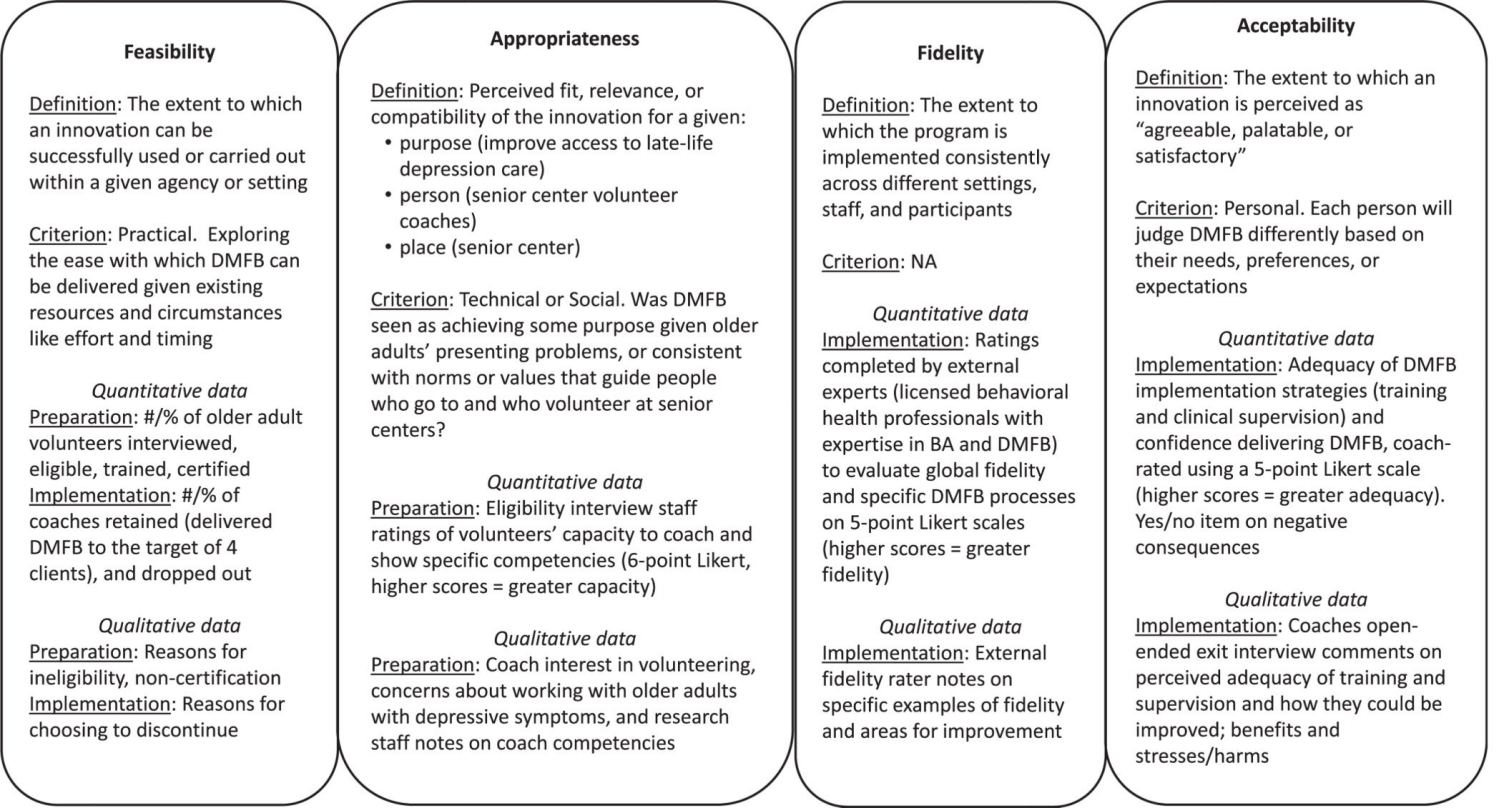
Implementation outcomes: Definitions, criteria^32^, implementation phase, data source. The definitions for implementation outcomes^21^ come from the Implementation Outcomes Framework. The criterion come from Weiner et al.’s 2017 paper.

These included feasibility, appropriateness, and acceptability, three constructs that reflect the fit of an evidence-based program or its implementation strategies to some criterion,^33^ and fidelity of volunteer coaches delivering behavioral activation as intended. Figure 1 provides details on implementation outcome definitions, criteria, and measures used. Implementation outcomes were assessed both during preparation (pre-implementation) and implementation phases.^22^

### Data Analysis

We used R^34^ for quantitative analysis and Excel for qualitative analysis. Quantitative analysis included descriptive statistics of quantitative indicators (i.e., frequency and percentage for categorical variables, mean and standard deviation for continuous variables). Qualitative data were analyzed via thematic analysis^35^ using the rapid framework method^36^ to understand key patterns in the data. Data was reduced with two coders using deductive coding with the IOF and inductive coding to allow additional themes to emerge that may not be captured in the IOF as it has not been applied extensively to older adults or community-based organizations. Discrepancies were resolved with the PIs and lead authors and member checking was done with community partners. We organized themes using interpretation memos and used joint display tables^37^ to integrate the qualitative and quantitative data to evaluate DMFB’s feasibility, appropriateness, fidelity and acceptability.

## RESULTS

Characteristics of eligible DMFB coaches (*N* = 45) are provided in Table 1. These 45 older adults had a mean age of 69.8 (SD = 6.3), 85.3% were women, 45.4% were people of color, 24.1% were low-income, and 53.1% lived alone.

### Feasibility

**FIGURE 2.**
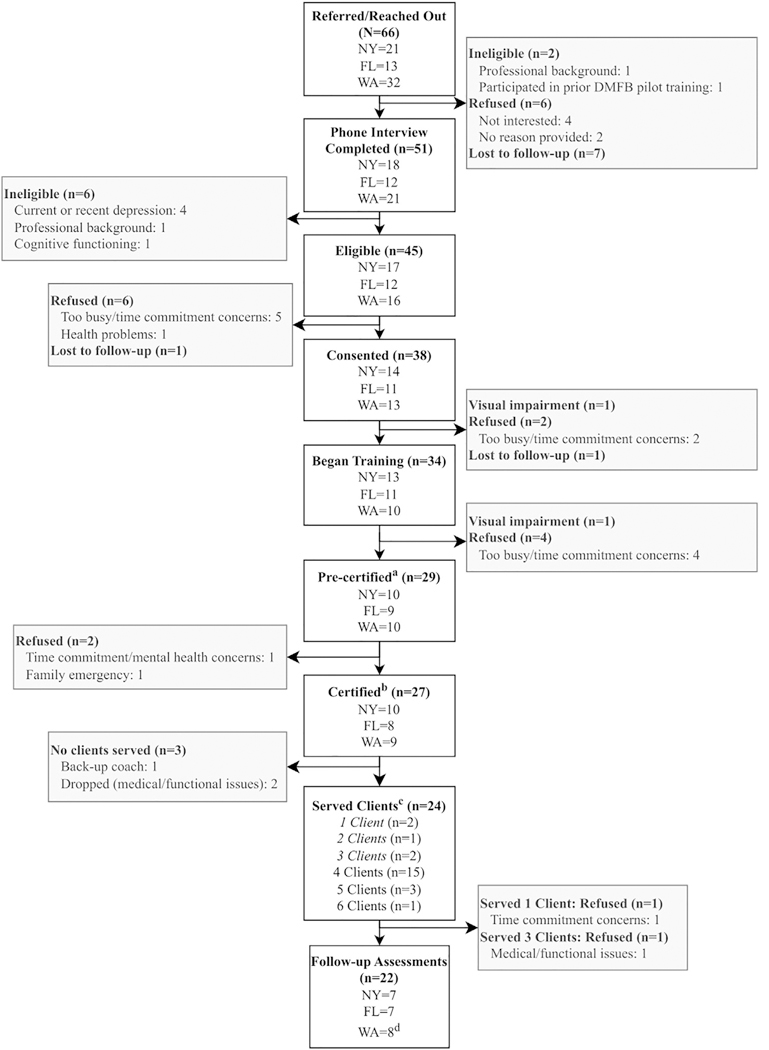
CONSORT diagram to show feasibility of DMFB implementation. ^a^Approved to see first “practice” case. ^b^Certified coaches successfully completed one “practice” case. ^c^The study design aimed for four RCT clients per coach. ^d^Includes one participant who provided partial follow-up assessment data when leaving the study after serving four clients.

Forty-five (68.2%) of 66 individuals interested in serving as a DMFB coach met eligibility criteria. Of the 66 interested volunteers, 34 (51.2%) were trained, 27 (40.1%) were certified as coaches following adequate fidelity working with an initial “practice” client, and 24 (36.4%) engaged 1 or more subsequent clients (Fig. 2). Among eligible coaches (N = 45), 34 (75.6%) began training, 27 (60.0%) were certified, and 24 (53.3) engaged at least one DMFB client. Reasons cited for noncertification or dropout were time commitment, co-occurring health issues (e.g. being hospitalized), caregiving duties, and no longer being interested.

### Appropriateness

Study investigators rated the 45 volunteers who met eligibility criteria to serve as coaches as having high capacity to coach (*M* = 5.3, SD = 0.8; Table 2). Reasons for ineligibility included lower interpersonal skills (*M* = 4.8, SD = 0.5), emotional stability and cognitive functioning (*M* = 3.5, SD = 1.0), commitment to serve as coach for 1 year (25% did not commit); and higher PHQ-9 (*M* = 9.1, SD = 3.1) and self-reported memory problems (50%). Ineligible coaches were rated as borderline capacity to serve as coaches (*M* = 3.5, SD = 1.0).

Qualitative data offers additional insights into volunteers’ fit as DMFB lay coaches. In the eligibility interviews, volunteers shared their experience helping and in service to others. Many were caregivers or had worked or volunteered in health-related fields − for example, one person led a Buddhist mediation group, and another managed a group home for people living with mental health conditions. Some coaches brought their lived experience managing mental health conditions (depression, anxiety, bipolar), which connected them to clients through their shared experience and provided hope and confidence in living well with depression. Volunteering with older adults was a way to give back to their community and help others. Volunteering also brought structure to their day which they were accustomed to in previous life roles working and raising families.

Coaches also brought skills in planning and organizing − they offered examples of ways they prioritized and accomplished tasks including how they used calendars or other tools to cue them, all skills that DMFB requires of both coaches and clients. Coaches also evidenced critical thinking skills from their work and family, managing diverse people in different situations and roles, which at times can be stressful. Similarly, coaches displayed good communication and conflict management skills (e.g., working with people with different viewpoints or that they did not get along with), and interpersonal skills such as empathy, compassion, and active listening.

### Fidelity

External global ratings from 52 randomly selected coach sessions ranged from “good” to “very good” *(M* = 4.62 of 5, SD = 0.65) overall and across the three sites, with only one session receiving a less than adequate fidelity rating (< 3; Table 3). Item-specific fidelity ratings (e.g., helping clients schedule rewarding daily activities) were also high.

Qualitative data from external raters for sessions with high ratings of 4 or 5 (N = 50) offer some insight into what coaches did well delivering DMFB to protocol. These sessions brought strong process skills, with coaches setting an agenda at the start of the session, administering the PHQ-9, discussing activities from the past week and reviewing successes and challenges with past week activity goals, supporting the participant in identifying and planning regular mood-boosting activities in the upcoming week, and wrapping up the session with reminders about upcoming homework.

Experts also rated coaches highly on their communication and interpersonal skills. They were warm, confident, knowledgeable, supportive, and empathetic to help address the low motivation and negative thinking that often accompanies depression. They managed time well to cover all agenda items while allowing space for clients to share their ideas. Coaches who were able to first engage the client well and get them to “buy into” the DMFB model subsequently had an easier time moving to the next step of helping clients list and plan activities. Coaches were observed to gently redirect clients back to the DMFB protocol when they started discussing extraneous topics.

Expert raters noted coaches’ strong “patient-centeredness,” allowing clients to guide activity planning and PHQ-9 rating of symptoms, and using clients’ own words and phrases to amplify their role in self-managing their depression. Coaches were also very attuned to the participant’s symptoms and how distress was impacting their ability to engage in the program and carry out planned activities. Being person-centered was particularly important for fidelity when clients were ambivalent about changing their behaviors or following the DMFB protocol.

Two sessions had lower fidelity ratings (one rated 2, one rated 3); here, coaches displayed poorer time management, deviated from the agenda during the PHQ-9 administration and activity planning, and provided direct advice rather than facilitating client independence in the process.

### Acceptability

During the exit interview, coaches who had served DMFB clients (N = 22) self-rated as highly confident delivering DMFB (*M* = 4.7, SD = 0.6; Table 4). Coaches also reported that both training (*M* = 4.5, SD = 0.6) and supervision (*M* = 4.6, SD = 0.5) were adequate on 5-point Likert scales.

From the qualitative portion of the exit interviews, coaches felt well-prepared by the training and materials provided, and they continued to feel supported by regular group supervision with a professional and other DMFB coaches; some coaches continued supervision after their responsibility ended to stay connected and maintain skills. Supervision brought “*comfort*”—providing a “*chance to check in, especially with difficult clients*” and a place to feel “*acknowledged and supported*” (White WA woman age 71). Some coaches desired more role plays and practice during or after the training or to shadow a coach to prepare them to deliver DMFB. Suggested ways to improve supervision include ensuring everyone can attend all sessions and better facilitating engagement from all coaches during sessions.

DMFB coaches described several ways they benefited from delivering DMFB. Coaches welcomed the satisfaction they got from helping others, seeing people make changes in their lives and get better—as one coach described, “*being part of someone*’*s happiness was incredibly gratifying*” (Black FL woman age 70). Coaches felt needed and valued because they were able to effectively help others. Furthermore, some coaches had lived with depression and had received help in the past, so doing DMFB offered a way for them to give back.

Coaches also liked applying the knowledge and skills they gained from being a DMFB coach to their own mental health—one coach appreciated how delivering DMFB helped them to learn about and understand more about late-life depression, recognizing “*the fact that it (depression) can happen to anyone at any time*” including herself [Black NY woman age 73]. Another coach shared how the client reminded them of the importance of DMFB’s rationale and skills —“*my last client mentioned these words to me:* ‘*I need to find my even keel.*’ *I learned what that meant and was able to apply it to my life*” [Latina FL woman age 60]. Coaches also realized they were not doing enough for themselves and would do more for self-care or other meaningful activities. Coaches welcomed how DMFB provided a way for them to keep their mind going, fill their day, and apply their education and skills towards something.

Lastly, coaches found the connection with clients appealing through the shared experiences of aging, and in some cases this bond continued after the program ended. Coaches found community in interacting with other older adults and having the “*opportunity to talk about mental health openly*” [white WA woman age 68]. The DMFB experience was a “*two-way street*” (Black NY woman age 73) in which coaches and clients connected over applying DMFB in their lives to feel better. As one coach put it, she could “*see herself in her clients*” [Black NY woman age 69], which allowed her to feel less alone, realize she needed to do things for herself, and both make changes in her life where she could while accepting that not all changes were possible.

Several factors lowered DMFB’s acceptability for coaches. One-third of coaches that completed exit surveys experienced some difficulty in their role. Qualitative interview data suggested that some of these unintended stresses were logistical - due to technology hurdles on the client side, or to challenges with scheduling both around their own and the client’s schedules. In addition, coaches described DMFB as less acceptable when they had poor rapport and struggled to get through to clients. In one instance, a coach talked about feeling they did not have enough knowledge or experience to adequately address a complex and challenging situation that a client was facing. Thus, providing DMFB can also have a negative emotional impact on some coaches.

## DISCUSSION

This study lends empirical support for the implementation of lay-delivered behavioral activation (DMFB) in senior centers that reach older adults historically underserved by mental health care. One in three older adults initially interested in serving as a coach were successfully trained, certified, and engaged as DMFB coaches, while other time commitments, caregiving duties, and health issues lowered the program’s feasibility. Trained older volunteers were appropriate for providing a mental health intervention to their peers due to their experience helping others and managing their own mental health, intervention delivery skills, and value of volunteering. DMFB coaches demonstrated high fidelity to the intervention, executing the protocol well while centering the needs of clients. In addition, coaches reported DMFB was acceptable, feeling very confident in delivering the program. They also found training and supervision to be adequate in their preparation and support, and suggested improvement with more role plays and ways to engage all coaches. Coaches felt satisfied helping and connecting with others and applying DMFB to their own mental health. DMFB was less acceptable when coaches experienced challenges with logistics, rapport, or working with clients with complex psychosocial issues. Implementation outcomes were similar for coaches across the three study locations regardless of different demographic characteristics.

These findings are consistent with previous research with Do More, Feel Better^19,38^ which found strong feasibility, fidelity, and acceptability, with volunteers increasing confidence over the course of the program. Other research with peer- and volunteer-led models has similar findings − for example, Woodard and colleagues^17^ reported comparable implementation outcomes in the context of a brief structured behavioral intervention for depression delivered by nonspecialists, while also pointing to needing to support managing coaches’ emotional investment in client’s success. Bryant and colleagues^39^ documented that volunteer-delivered BA was feasible and acceptable for treating depression in residential care facilities, with volunteers benefiting from helping others and gaining skills from the program.

Our study also suggests opportunities to improve the delivery of DMFB and other lay led mental health programs in real-world social service settings. Feasibility can be improved by making ineligibility criteria more prominent when outreaching to volunteers and building in flexibility to accommodate lay coaches’ competing demands. Appropriateness can be strengthened by including the DMFB study interview guide in the DMFB practice manual so sites can use this to gauge experiences and skills that are a good fit for DMFB − experience helping others, skills planning and organizing, and the value of giving back. To increase acceptability, we recommend highlighting the benefits that made DMFB acceptable identified in this study - social connection, tools to support your mental health, satisfaction from helping others − to engage new DMFB coaches. It will also be helpful to build in supports for the things that make DMFB less acceptable, such as challenges using technology and scheduling clients, and learning strategies to work with clients where rapport is lacking or clients are facing multiple complex life situations. Lastly, fidelity can be maximized by building training and supervision content on strategies for time management, agenda setting and sticking, and client-driven rather than advice-giving communication.

While this study provides strong evidence that older adult volunteers can be successful lay DMFB coaches, engaging and sustaining diverse coaches is hindered by older adults’ competing demands like health and caregiving issues and the need for ongoing recruitment for this short-term volunteer workforce. This points to the need for better supports which we will evaluate in our next implementation study to evaluate the effectiveness and costs of training, supervision, and other implementation strategies to support this workforce that reaches underserved older adults via senior centers and other community-based organizations.

This study brings several strengths and contributions to the literature. Our research adds to the dearth of implementation science studies in community-based social service settings and with older adults,^20^ and applying the Implementation Outcomes Framework to this population identified areas where DMFB delivery is effective and opportunities for improvement. Furthermore, conducting mixed methods research in the context of implementation and centering implementers’ perspectives^24^ are key for guiding future adaptations and logic models to strengthen implementation research and practice for healthy aging.^40^ Our small sample size may limit generalizability to other contexts. Furthermore, additional research is needed to determine whether these implementation outcomes hold for DMFB coaches outside an RCT study context.

In summary, this study suggests older adult volunteers can effectively deliver quality streamlined BA for late-life depression. Our learnings can also guide future research and practice with lay-led interventions. DMFB is promising for closing the mental health care gap for older adults where they live, work, play, pray and age.

## Supplementary Material

Supplement 1. Interview Guides

Supplementary material associated with this article can be found, in the online version, at doi:10.1016/j.osep.2025.05.001.

## Figures and Tables

**TABLE 1.** Participant Characteristics (Eligible DMFB Coaches) (N = 45)

	Eligible (*n* = 45)	Trained, Not Certified (*n* = 7)	Certified (*N* = 24)	FL (*n* = 7)	NY (*n* = 9)	WA (*n* = 8)

Age, *M* (SD)	69.8 (6.4)	67.3 (8.3)	69.9 (6.3)	67.1 (7.4)	73.8 (5.5)	67.6 (3.4)
*n*	37	5	23	7	9	7

Gender^a^						
*n*	34	5	24	7	9	8
Woman	29 (85.3%)	4 (80%)	21 (87.5%)	7 (100%)	8 (88.9%)	6 (75%)
Man	5 (14.7%)	1 (20%)	3 (12.5%)	0	1 (11.1%)	2 (25%)

Race						
*n*	33	4	24	7	9	8
White	18 (54.6%)	1 (25%)	14 (58.3%)	4 (57.1%)	3 (33.3%)	7 (87.5%)
Black or African American	11 (33.3%)	2 (50%)	7 (29.2%)	2 (28.6%)	4 (44.4%)	1 (12.5%)
Asian or Pacific Islander	1 (3%)	0	1 (4.2%)	0	1 (11.1%)	0
Native American or Alaskan Native	0	0	0	0	0	0
Other^b^	3 (9.1%)^d^	1 (25%)^d^	2 (8.3%)	1 (14.3%)	1 (11.1%)	0

Ethnicity						
*n*	33	4	24	7	9	8
Yes	4 (12.1%)	1 (25%)	2 (8.3%)	2 (28.6%)	0	0
No	29 (87.9%)	3 (75%)	22 (91.7%)	5 (71.4%)	9 (100%)	8 (100%)

Nativity						
*n*	32	4	23	7	8	8
United States	28 (87.5%)	4 (100%)	19 (82.6%)	6 (85.7%)	5 (62.5%)	8 (100%)
Another country^c^	4 (12.5%)	0	4 (17.4%)	1 (14.3%)	3 (37.5%)	0

Marital Status						
*n*	32	4	24	7	9	8
Married or living as married	11 (34.4%)	2 (50%)	7 (29.2%)	2 (28.6%)	1 (11.1%)	4 (50%)
Widowed	4 (12.5%)	1 (25%)	3 (12.5%)	0	1 (11.1%)	2 (25%)
Separated	0	0	0	0	0	0
Divorced	7 (21.9%)	0	6 (25%)	2 (28.6%)	2 (22.2%)	2 (25%)
Single or never married	8 (25%)	1 (25%)	6 (25%)	2 (28.6%)	4 (44.4%)	0
Refused	1 (3.1%)	0	1 (4.2%)	0	1 (11.1%)	0
Not asked	1 (3.1%)	0	1 (4.2%)	1 (14.3%)	0	0

Living situation						
*n*	32	4	24	7	9	8
Alone	17 (53.1%)	2 (50%)	13 (54.2%)	5 (71.4%)	6 (66.7%)	2 (25%)
With others	14 (43.8%)	2 (50%)	10 (41.7%)	2 (28.6%)	3 (33.3%)	5 (62.5%)
Refused	1 (3.1%)	0	1 (4.2%)	0	0	1 (12.5%)

Years of schooling, *M* (SD)	16.6 (2.8)	16.7 (1.2)	16.5 (3.1)	16.4 (4.5)	15.2 (2.4)	17.9 (1.6)
*n*	31	3	24	7	9	8

Income						
*n*	29	3	23	7	8	8
<$17,599	1 (3.4%)	0	1 (4.3%)	1 (14.3%)	0	0
$17,600−$31,999	6 (20.7%)	1 (33.3%)	5 (21.7%)	2 (28.6%)	3 (37.5%)	0
$32,000−$74,999	6 (20.7%)	1 (33.3%)	4 (17.4%)	1 (14.3%)	2 (25%)	1 (12.5%)
$75,000 or more	6 (20.7%)	0	4 (17.4%)	0	1 (12.5%)	3 (37.5%)
Don’t know	2 (6.9%)	0	2 (8.7%)	1 (14.3%)	1 (12.5%)	0
Refused	8 (27.6%)	1 (33.3%)	7 (30.4%)	2 (28.6%)	1 (12.5%)	4 (50%)

Currently working						
*n*	32	4	24	7	9	8
Yes	2 (6.3%)	0	1 (4.2%)	1 (14.3%)	0	0
No	29 (90.6%)	4 (100%)	22 (91.7%)	6 (85.7%)	9 (100%)	7 (87.5%)
Refused	1 (3.1%)	0	1 (4.2%)	0	0	1 (12.5%)

Currently on Medicaid						
*n*	30	3	23	7	8	8
Yes	4 (13.3%)	0	3 (13%)	2 (28.6%)	1 (12.5%)	0
No	22 (73.3%)	2 (66.7%)	17 (73.9%)	4 (57.1%)	7	6 (75%)
Don’t know	1 (3.3%)	0	1 (4.3%)	0	0	1 (12.5%)
Refused	3 (10%)	1 (33.3%)	2 (8.7%)	1 (14.3%)	0	1 (12.5%)

a Original response options for gender identity were “female” and “male,” and are reported as “woman” and “man,” respectively.

b One participant from FL specified “Jewish.”

c One participant from NY specified “Israel.” One participant from FL specified “Nassau (Bahamas).”

d One participant selected “NA.”

**TABLE 2.** Appropriateness (Older Adults Interviewed for Eligibility) (N = 45)

	Eligible (*N* = 45)	FL (*n* = 12)	NY (*n* = 17)	WA (*n* = 16)	Ineligible (*n* = 6)

Interpersonal skills, *n*	44	11	17	16	4
*M* (SD)	5.3 (0.6)	5.3 (0.6)	5.4 (0.5)	5.3 (0.6)	4.8 (0.5)

Emotional stability and cognitive functioning, *n*	44	12	17	15	4
*M* (SD)	5.5 (0.7)	5.3 (0.5)	5.5 (0.9)	5.5 (0.5)	3.5 (1.0)

Volunteer capacity to serve as coach, *n*	45	12	17	16	4
*M* (SD)	5.3 (0.8)	5.5 (0.7)	5.1 (0.9)	5.3 (0.6)	3.5 (1.0)

Commitment to serve as coach to see 5 clients over 1 year, *n* (%)	42/43 (97.7%)	11/11 (100%)	16/16 (100%)	15/16 (93.8%)	3/4 (75%)

Comfort with confidentiality, *n* (%)	45/45 (100%)	12/12 (100%)	17/17 (100%)	16/16 (100%)	4/4 (100%)

PHQ-9, *n*	24	7	12	5	3
*M* (SD); score for *n* = 1	1.3 (1.4)	0.7 (1.1)	1.4 (1.5)	2.0 (1.4)	9.7 (3.1)

Did not meet criteria for memory and cognition screen (<3), *n* (%)	0	0	0	0	0

Past mental health treatment, *n* (%)	23/42 (54.8%)	7/12 (58.3%)	7/14 (50%)	9/16 (56.3%)	2/4 (50%)

Past mental health hospitalization, *n* (%)	2/41 (4.9%)	0/12 (0%)	1/13 (7.7%)	1/16 (6.3%)	1/4 (25%)

History of psychosis, *n* (%)	1/40 (2.5%)	0/12 (0%)	1/13 (7.7%)	0/15 (0%)	0/4 (0%)

Self-reported memory problems, *n* (%)	5/40 (12.5%)	3/10 (30%)	2/14 (14.3%)	0/16 (0%)	2/4 (50%)

**Table 3.** Fidelity Ratings^a^ from DMFB Coach sessions (N=52 sessions).

Quantitative Mean(SD) Fidelity Rating ^b^		Qualitative Fidelity Reviewer Feedback
		Higher fidelity ratings (4 or 5):
Global Fidelity Rating	4.62(0.65)	Process skills (e.g. time management): *Excellent session. Coach set and kept to an agenda, explored the past week in depth, and asked detailed questions about activities participant might want to do.*
	
Reviewed Progress (PHQ-9, Planned Activities)	4.63 (0.67)	Communication and interpersonal skills, e.g. empathetic: *Another thoughtful and thorough session. Participant had struggled a little with motivation and negative thinking in past week. Coach provided helpful suggestions to keep momentum going on enjoyable activities and think ahead about how to overcome barriers in next week.*
	
Scheduled Activities	4.66 (0.72)	Person-centered: *This was a slightly more difficult than average session, as the participant is engaged in many activities, although not fulfilled, and is not bought into the idea that adding any more activities will improve depression. Coach did a good job meeting the participant where she was at, rolling with resistance, and encouraging participant to articulate changes she wanted to make.*
	
Concluded Session + Homework	4.52 (0.82)	Engaged participant: *Great second session! Excellent rapport. Coach is well-organized and clearly has materials/questions together to guide session in a responsive way. This participant was very receptive, so is possible coach may experience new challenges with a participant with more barriers to action.*
	
		Lower fidelity ratings (2 or 3):
Process Tasks	4.54 (0.82)	Poor time management and not being person-centered: *The PHQ-9 took 11 minutes to complete…It would be helpful to set a verbal agenda at the start of the session, to keep on track with completing session items…No activity form was formally reviewed, though there was a general discussion of her week. The agenda for the DMFB intervention was not followed.*

Communication & Interpersonal Skills	4.62 (0.63)	

a External fidelity ratings were conducted for 52 coach sessions.

b Scale of 1 to 5, with 5 being highest rating

**TABLE 4.** Acceptability (DMFB Certified Coaches) (N=22)^c^

	Overall (*N* = 22)	FL (*n* = 7)	NY (*n* = 7)	WA (*n* = 8)

Volunteer questionnaire, *n*^a^	21	7	7	7
Coach training, *M* (SD)	4.5 (0.6)	4.6 (0.5)	4.4 (0.5)	4.4 (0.8)
Coach supervision, *M* (SD)	4.6 (0.5)	4.6 (0.5)	4.6 (0.5)	4.7 (0.5)
Confidence, *M* (SD)	4.7 (0.6)	4.7 (0.5)	4.7 (0.5)	4.6 (0.8)
Negative consequences, *n* (%)	6/18 (33.3%)	2/7 (28.6%)	2/7 (28.6%)	2/4^b^ (50%)

a Range = 1−5.

b Includes one participant who provided a response for negative consequences when leaving the study; this participant did not complete the volunteer questionnaire.

c Two of the 24 DMFB coaches did not complete follow-up assessment data.

## Highlights

### What is the primary question addressed by this study?

Can simplified behavioral activation (“Do More, Feel Better”) be delivered successfully by older adult lay volunteer coaches?

### What is the main finding of this study?

The lay volunteer coaches were able to deliver the program with fidelity and they found it feasible, appropriate, and acceptable.

### What is the meaning of the finding?

These findings add to limited literature on implementation outcomes for lay-led community-based interventions for older adults and document successful implementation.

## References

[1] World Health Organization (WHO). Mental health. https://www.who.int/news-room/fact-sheets/detail/mental-health-strengthening-our-response. Acccessed May 28, 2025

[2] Zenebe Y, Akele B, W/Selassie M, : Prevalence and determinants of depression among old age: a systematic review and meta-analysis. Ann Gen Psychiatry 2021; 20(1):55;doi:10.1186/s12991-021-00375-x 34922595 PMC8684627

[3] Jimenez DE, Park M, Rosen D, : Centering culture in mental health: differences in diagnosis, treatment, and access to care among older people of color. Am J Geriatr Psychiatr 2022; 30 (11):1234–1251;doi:10.1016/j.jagp.2022.07.001 PMC9799260 35914985

[4] Bureau of Health Workforce, Health Resources and Services Administration (HRSA) USD of H& HS. Designated Health professional shortage Areas statistics: designated HPSA quarterly summary. https://data.hrsa.gov/topics/health-workforce/shortage-areas. 2023. Acccessed May 28, 2025

[5] Deferio JJ, Breitinger S, Khullar D, : Social determinants of health in mental health care and research: A case for greater inclusion. J Am Med Informat Associat 2019; 26(8–9):895–899; doi:10.1093/jamia/ocz049 PMC6696493 31329877

[6] Raue PJ, Sirey JA, Gum A, : Protocol for a collaborative randomised effectiveness trial of lay-delivered versus clinician-delivered behavioural activation in senior centres. BMJ Open 2022; 12(8):e066497;doi:10.1136/bmjopen-2022-066497 PMC9403148 35998966

[7] Raue PJ, Sershinhe K: Mental health task sharing: training volunteers, peers, and interns. Generations 2024; 48:1–9

[8] National Council on Aging: Get the facts on senior centers. https://www.ncoa.org/article/get-the-facts-on-senior-centers/; 2024 Accessed February 7, 2025

[9] Ochieng N, Cubanski J, Neuman T, : How many older adults live in poverty? Kaiser family foundation issue brief; https://www.kff.org/medicare/issue-brief/how-many-older-adults-live-in-poverty/ Accessed March 5, 2025

[10] Corporation for National and Community Service: Senior volunteering at a 10-year high. Philanthropy News Digest 2013

[11] Kim ES, Whillans AV, Lee MT, : Volunteering and subsequent health and well-being in older adults: an outcome-wide longitudinal approach. Am J Prev Med 2020; 59(2):176–186;doi:10.1016/j.amepre.2020.03.004 32536452 PMC7375895

[12] Nichol B, Wilson R, Rodrigues A, : Exploring the effects of volunteering on the social, mental, and physical health and well-being of volunteers: an Umbrella Review. Voluntas 2024; 35 (1):97–128;doi:10.1007/s11266-023-00573-z PMC10159229 37360509

[13] Dias A, Azariah F, Anderson SJ, : Effect of a lay counselor intervention on prevention of major depression in older adults living in low- and middle-income countries. JAMA Psychiatry 2019; 76(1):13;doi:10.1001/jamapsychiatry.2018.3048 30422259 PMC6583466

[14] Weaver A, Lapidos A: Mental health interventions with Community Health workers in the United States: a systematic review. J Health Care Poor Underserved 2018; 29(1):159–180; doi:10.1353/hpu.2018.0011 29503292

[15] Barnett ML, Gonzalez A, Miranda J, : Mobilizing community health workers to address mental health disparities for underserved populations: A systematic review. Administrat Pol Ment Health Mental Health Serv Res 2018; 45(2):195–211; doi:10.1007/s10488-017-0815-0 PMC5803443 28730278

[16] Lam AHK, Yeung DY, Chung EKH: Benefits of volunteerism for middle-aged and older adults: comparisons between types of volunteering activities. Ageing Soc 2023; 43(10):2287–2306; doi:10.1017/S0144686X21001665

[17] Woodard GS, Mraz A, Renn BN: Perspectives of nonspecialists delivering a brief depression treatment in the United States: a qualitative investigation. BMC Psychiatry 2023; 23(1):32; doi:10.1186/s12888-023-04528-y 36639746 PMC9839228

[18] Russell AR, Storti MAH, Handy F: Volunteer retirement and well-being: evidence from older adult volunteers. Int J Community Well-Being 2022; 5(2):475–495;doi:10.1007/s42413-021-00157-z

[19] Raue PJ, Hawrilenko M, Corey M, : Do more, feel better”: pilot RCT of lay-delivered behavioral activation for depressed senior center clients. Behav Ther 2022; 53(3):458–468;doi:10.1016/j.beth.2021.11.005 35473649 PMC9046684

[20] Proctor EK, Bunger AC, Lengnick-Hall R, : Ten years of implementation outcomes research: a scoping review. Implementat Sci 2023; 18(1):31;doi:10.1186/s13012-023-01286-z PMC10367273 37491242

[21] Proctor E, Silmere H, Raghavan R, : Outcomes for implementation research: conceptual distinctions, measurement challenges, and research agenda. Administrat Pol Ment Health Mental Health Serv Res 2011; 38(2):65–76;doi:10.1007/s10488-010-0319-7 PMC3068522 20957426

[22] Aarons GA, Hurlburt M, Horwitz SM: Advancing a conceptual model of evidence-based practice implementation in public service sectors. Administrat Pol Ment Health Mental Health Serv Res 2011; 38(1):4–23;doi:10.1007/s10488-010-0327-7 PMC3025110 21197565

[23] Schoonenboom J, Johnson RB: How to construct a mixed methods research design. KZfSS Kölner Zeitschrift für Soziologie und Sozialpsychologie 2017; 69(S2):107–131;doi:10.1007/s11577-017-0454-1 28989188 PMC5602001

[24] Palinkas LA, Aarons GA, Horwitz S, : Mixed method designs in implementation research. Administrat Pol Ment Health Mental Health Serv Res 2011; 38(1):44–53;doi:10.1007/s10488-010-0314-z PMC3025112 20967495

[25] Cuijpers P, Karyotaki E, Harrer M, : Individual behavioral activation in the treatment of depression: A meta analysis. Psychother Res 2023; 33(7):886–897;doi:10.1080/10503307.2023.2197630 37068380

[26] Powell BJ, Waltz TJ, Chinman MJ, : A refined compilation of implementation strategies: results from the Expert Recommendations for implementing Change (ERIC) project. Implementat Sci 2015; 10(1):21;doi:10.1186/s13012-015-0209-1 PMC4328074 25889199

[27] Leeman J, Birken SA, Powell BJ, : Beyond “implementation strategies”: classifying the full range of strategies used in implementation science and practice. Implementat Sci 2017; 12 (1):125;doi:10.1186/s13012-017-0657-x PMC5670723 29100551

[28] Kroenke K, Spitzer RL, Williams JBW: The PHQ-9. J Gen Intern Med 2001; 16(9):606–613;doi:10.1046/j.1525-1497.2001.016009606.x 11556941 PMC1495268

[29] Callahan CM, Unverzagt FW, Hui SL, : Six-item screener to identify cognitive impairment among potential subjects for clinical research. Med Care 2002; 40(9):771–781;doi:10.1097/00005650-200209000-00007 12218768

[30] Hamilton M: A rating scale for depression. J Neurol Neurosurg Psychiatry 1960; 23(1):56–62;doi:10.1136/jnnp.23.1.56 14399272 PMC495331

[31] Brandt J, Spencer M, Folstein M: Telephone interview for cognitive status. PsycTESTS Dataset 2022;doi:10.1037/t28542-000

[32] Harris PA, Taylor R, Minor BL, : The REDCap consortium: building an international community of software platform partners. J Biomed Inform 2019; 95:103208;doi:10.1016/j.jbi.2019.103208 31078660 PMC7254481

[33] Weiner BJ, Lewis CC, Stanick C, : Psychometric assessment of three newly developed implementation outcome measures. Implementation Science 2017; 12(1):108;doi:10.1186/s13012-017-0635-3 28851459 PMC5576104

[34] R Core Team: R: A language and environment for statistical computing. https://www.R-project.org/; 2023 Accessed February 7, 2025

[35] Nowell LS, Norris JM, White DE: Moules NJ. Thematic analysis. Int J Qual Methods 2017; 16(1);doi:10.1177/1609406917733847

[36] Gale RC, Wu J, Erhardt T, : Comparison of rapid vs in-depth qualitative analytic methods from a process evaluation of academic detailing in the Veterans Health Administration. Implementation Sci 2019; 14(1):1–12;doi:10.1186/s13012-019-0853-y PMC6359833 30709368

[37] Guetterman TC, Fetters MD, Creswell JW: Integrating quantitative and qualitative results in health science mixed methods research through joint displays. Ann Fam Med 2015; 13(6):554–561;doi:10.1370/afm.1865 26553895 PMC4639381

[38] Raue PJ, Dawson A, Hoeft T, : Acceptability of a lay-delivered intervention for depression in senior centers. Aging Ment Health 2021; 25(3):445–452;doi:10.1080/13607863.2019.1698515 31799880 PMC7269871

[39] Bryant C, Brown L, Polacsek M, : Volunteer-led behavioural activation to reduce depression in residential care: a feasibility study. Pilot Feasibility Stud 2020; 6(1):95;doi:10.1186/s40814-020-00640-y 32670597 PMC7341647

[40] Estabrooks PA, Glasgow RE: Developing a dissemination and implementation research agenda for aging and public health: the what, when, how, and why? Front Public Health 2023; 11; doi:10.3389/fpubh.2023.1123349 PMC9939692 36815160

