# Supplementary material for: Older Adult Volunteers’ Experiences Delivering a Lay-Led Behavioral Activation Program for Depression Among Community-Dwelling Older Adults: A Mixed Methods Study
Source: Am J Geriatr Psychiatry Open Sci Educ Pract. Author manuscript; Available in PMC 2025 Dec 16. (PMC12704987; doi:10.1016/j.osep.2025.05.001)
Supplement: Supplement 1. Interview Guides [file NIHMS2089043-supplement-Supplement_1__Interview_Guides.pdf]

Supplement 1. Do More, Feel Better Volunteer Interview Guides

A. Volunteer Coach Eligibility Interview Guide for Do More, Feel Better Program

(chat for a couple minutes, observe interpersonal skills)

Thank you for meeting with me today. The purpose of this interview is to learn more about your experiences and skills, to make sure that this Volunteer Coach role will be a good fit for you. Before I begin with the questions, I'll briefly review the program again.

The "Do More, Feel Better" program involves meeting with a center member whom we have identified as having significant symptoms of depression, for example feeling down and not able to take pleasure in usual activities for an extended period of time. We will refer any member experiencing significant symptoms of depression for mental health care from their primary care physician or a mental health specialist. But we also feel that meeting with volunteer members who have been trained in this program can help them take care of themselves and get back to their usual selves.

The "Do More, Feel Better" program involves establishing a warm and friendly, but professional relationship with these members; discussing their symptoms of depression with them and the impact on their quality of life; helping them explore enjoyable and rewarding activities that they have stopped doing; helping them schedule one activity a day that they would be interested in pursuing; and reviewing their efforts and providing ongoing support for their goals.

Training involves meeting with us every week for 4 weeks, learning about depression in older adults, and learning how to be a volunteer peer for this program.

If you become certified, we provide close supervision when you meet with members for the program.

Do you have any questions for me before we begin with the questions?

We ask all the same questions of each person who is interested in becoming a Volunteer Coach. Some questions ask about your experiences and skills helping others, and some questions ask about personal experiences and skills that are important for being a Volunteer Coach. It's ok to be brief with your answers, and I will ask for more details if needed.

Competency #1: Experience/Service to Others

Tell me about your education, your life, and your work and volunteer experience.

How do you feel this experience may have prepared you to be a volunteer coach working with other members who experience depression?

Can you give an example of how your experience has helped you to help another person going through depression, or having a difficult time emotionally?

Competency #2: Communication

Can you give an example of a time when you communicated successfully with another person, even when that individual may not have personally liked you (or a person that you may not have personally liked)?

Rate volunteer's overall interpersonal skills:

☐ Very Poor ☐ Poor ☐ Borderline ☐ Satisfactory ☐ Good ☐ Very Good

Competency #3: Planning and Organizing

How do you schedule your time and set priorities? How do you handle several responsibilities at once? Can you give me a specific example of a time when you did this?

Competency #4: Critical Thinking

Tell me about a time when you had to solve a problem with very little guidance or direction.

Rate volunteer's overall ability to follow the structure of the "Do More, Feel Better program":

☐ Very Poor ☐ Poor ☐ Borderline ☐ Satisfactory ☐ Good ☐ Very Good

The next questions may seem a little personal. We ask about any personal experiences with emotional difficulties or mental health care. It's ok to have had these personal experiences; the main issue is making sure that Volunteer Coaches are at a time in their life when they can learn the program and focus on helping and supporting others.

Competency #5: Emotional Stability and Cognitive Functioning

1. *Do you have any history of psychiatric or emotional problems? If yes, can you tell me how that is affecting you now?*
   ☐Yes ☐No ☐NA (-7) ☐Don't Know (-8) ☐Refused (-9)
   [Record Comments/Questions]

   Notes:

2. *Have you ever received any kind of treatment for psychiatric or emotional problems? If so, what?*
   ☐Yes ☐No ☐NA (-7) ☐Don't Know (-8) ☐Refused (-9)
   [Record Comments/Questions]

Notes:

3. *Have you ever been hospitalized for psychiatric or emotional problems?*
   ☐Yes ☐No ☐NA (-7) ☐Don't Know (-8) ☐Refused (-9)
   [Record Comments/Questions]

   Notes:

4. *Now I am going to ask you about unusual experiences that people sometimes have. Have you been bothered by hearing things that other people couldn't hear, such as voices even when no one was around? Or feeling that someone could hear your thoughts, or that you could hear what another person was thinking?*
   ☐Yes ☐No ☐NA (-7) ☐Don't Know (-8) ☐Refused (-9)

   [Record Comments/Questions]

   Notes:

5. *Have you been bothered by problems with memory (for example, learning new information) or with location (for example, finding your way home)?*
   ☐Yes ☐No ☐NA (-7) ☐Don't Know (-8) ☐Refused (-9)
   [Record Comments/Questions]

   Notes:

6. *Administer the Brief 6-Item Memory Cognition Screen for cognitive functioning: MCS Score:*

Rate volunteer's overall emotional stability and cognitive functioning:

☐ Very Poor ☐ Poor ☐ Borderline ☐ Satisfactory ☐ Good ☐ Very Good

Additional Questions:

1. *What appeals to you about volunteering for the "Do More, Feel Better" program?*

2. *Do you have any concerns about working with a senior center member who has depression?*
   ☐Yes ☐No ☐NA (-7) ☐Don't Know (-8) ☐Refused (-9)
   [Record Comments/Questions]

3. *If you were trained up in the program, would you be able to commit to meeting with 4-5 clients during your participation? You'll meet each client weekly for 30-45 minute sessions over the course of 9 weeks.*
   ☐Yes ☐No ☐NA (-7) ☐Don't Know (-8) ☐Refused (-9)
   [Record Comments/Questions]

4. *Would you be comfortable keeping any information the client reveals to you as confidential, just shared with your supervisor?*
   ☐Yes ☐No ☐NA (-7) ☐Don't Know (-8) ☐Refused (-9)
   [Record Comments/Questions]

5. *Do you have any questions for me?*

Now that we have finished, I'll review the next steps. I talk with the supervisors after each interview to decide whether each person is a good fit for the program. If so, then I will get back in touch with you within a few days. There is a document we would need to review, called a consent form, to review all the details of the project and make sure you agree. Also, you would need to get a background check, which Hillsborough County Aging Services requires. We will help to set up all these things. Once we have identified all the volunteers, we will work on scheduling the training. Once again, thank you so much for your interest and your time today!

Final Rating: Rate Volunteer's Overall Capacity to Serve as a Volunteer Coach:

☐ Very Poor ☐ Poor ☐ Borderline ☐ Satisfactory ☐ Good ☐ Very Good

B. Volunteer Coach Exit Interview Guide for Do More, Feel Better Program

The purpose of this interview is to assess your experiences as a volunteer coach for the "Do More, Feel Better" program. Please complete the following survey by checking the responses to all questions even if you are unsure of the best answer. We will also ask you some follow-up open-ended questions as well to hear your perspectives about the program. We appreciate your participation.

Please rate your level of agreement with the following statements:

I had adequate training in the "Do More, Feel Better" program before I saw my first depressed client.

- ❑ (1) Strongly disagree
- ❑ (2) Disagree
- ❑ (3) Neither agree nor disagree
- ❑ (4) Agree
- ❑ (5) Strongly agree

What was most helpful about the training?

____

What if anything could improve the training?

____

I received adequate ongoing support and supervision in my work as a coach.

- ❑ (1) Strongly disagree
- ❑ (2) Disagree
- ❑ (3) Neither agree nor disagree
- ❑ (4) Agree
- ❑ (5) Strongly agree

What was most helpful about the ongoing supervision?

____

What if anything could improve the ongoing supervision?

____

I am confident that I can continue to conduct the “Do More, Feel Better” program with depressed clients, with regular supervision.

- ❑ (1) Strongly disagree
- ❑ (2) Disagree
- ❑ (3) Neither agree nor disagree
- ❑ (4) Agree
- ❑ (5) Strongly agree

What is the primary benefit you have received from this program?

____

____

____

____

Have you experienced any other personal benefits from serving as a volunteer coach for this program?

____

____

____

____

Have you experienced anything negative as a result of serving as a volunteer coach? Yes/No:

If yes, please explain:

____

____

____

____

Thank you for your time and participation!
